# Deep Learning Based One-Class Detection System for Fake Faces Generated by GAN Network

**DOI:** 10.3390/s22207767

**Published:** 2022-10-13

**Authors:** Shengyin Li, Vibekananda Dutta, Xin He, Takafumi Matsumaru

**Affiliations:** 1 Graduate School of Information, Production and Systems, Waseda University, Kitakyushu 808-0135, Japan; 2 Institute of Micromechanics and Photonics, Faculty of Mechatronics, Warsaw University of Technology, 00-661 Warszawa, Poland

**Keywords:** Multi-Channel Convolutional Neural Network (MCCNN), face anti-spoofing, attention learning, self-attention, data augmentation, deep learning, Generative Adversarial Network (GAN), Weakly Supervised Learning (WSL)

## Abstract

Recently, the dangers associated with face generation technology have been attracting much attention in image processing and forensic science. The current face anti-spoofing methods based on Generative Adversarial Networks (GANs) suffer from defects such as overfitting and generalization problems. This paper proposes a new generation method using a one-class classification model to judge the authenticity of facial images for the purpose of realizing a method to generate a model that is as compatible as possible with other datasets and new data, rather than strongly depending on the dataset used for training. The method proposed in this paper has the following features: (a) we adopted various filter enhancement methods as basic pseudo-image generation methods for data enhancement; (b) an improved Multi-Channel Convolutional Neural Network (MCCNN) was adopted as the main network, making it possible to accept multiple preprocessed data individually, obtain feature maps, and extract attention maps; (c) as a first ingenuity in training the main network, we augmented the data using weakly supervised learning methods to add attention cropping and dropping to the data; (d) as a second ingenuity in training the main network, we trained it in two steps. In the first step, we used a binary classification loss function to ensure that known fake facial features generated by known GAN networks were filtered out. In the second step, we used a one-class classification loss function to deal with the various types of GAN networks or unknown fake face generation methods. We compared our proposed method with four recent methods. Our experiments demonstrate that the proposed method improves cross-domain detection efficiency while maintaining source-domain accuracy. These studies show one possible direction for improving the correct answer rate in judging facial image authenticity, thereby making a great contribution both academically and practically.

## 1. Introduction

### 1.1. Fake Image Generation Technology

In response to rapidly developing fake image generation technology, existing researchers have developed/introduced many effective methods and discussed various problems encountered in the research and development process. The purpose of digital image forensics research is to identify a forged image to avoid damage caused by the image. In this paper, the main purpose is to detect fake images generated by Generative Adversarial Networks; therefore, we discuss the recent developments these networks below in some detail.

Most detection algorithms for fake faces are performed at the pixel level. As this problem is essential for image analysis and works under the umbrella of binary classification, using a conventional Convolutional Neural Network (CNN) is a practical approach. This method uses a high-pass filter to preprocess the image and then a five-layer neural network to train the processed data (2018) [1]. Although it is a simple CNN model, its performance is good enough for recognizing fake images with homology problems. However, a major drawback is the fragility of the method. To counter this problem, Cozzolino et al. (2018) [2] proposed a new network structure containing two fully connected layers that act as the encoder and decoder for the downsampling and upsampling functions, respectively. The fundamental principle behind this approach is the difference between real and fake images after downsampling and upsampling, however, the fatal flaw of this method is that it cannot satisfy the detection of larger images. Quan et al. (2018) [3] introduced an approach for detecting the presence of subtle generative traces in fake images by cropping large images that are difficult to train and feeding them into a neural network. Later, they cropped the images again to a fixed size during the neural network transfer process, then used a voting mechanism to determine the authenticity of the images.

Goodfellow et al. (2014) [4] proposed a new training model for unsupervised learning, the so-called Generative Adversarial Networks (GANs). The other proposed model is the discriminative model. One of the ideas mentioned in the above paper is that when the performance of both the generative model and the discriminative model is good enough, the generative model must beat the discriminative model, which is the convergence of the generative adversarial network. Initially, GAN networks can only generate images of unknown meaning.

**Figure 1:**
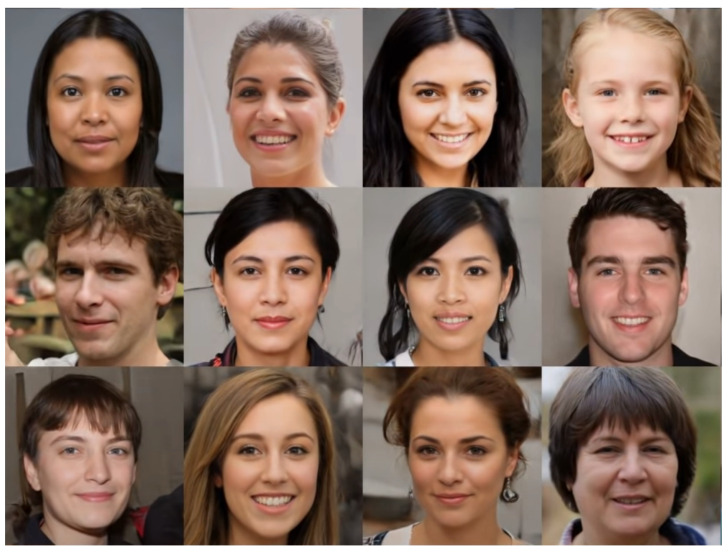
High-quality face photos generated by GAN [8].

Subsequently, GAN networks have seen considerable development thanks to their significance in forensic science. Radford et al. (2015) [5] first considered a GAN network using a deep neural network structure, called a Deep Convolutional Generative Adversarial Network (DCGAN). The same consideration was taken into account for extending the CNN network. To accomplish this task, the generator of the GAN network uses the ReLU (Rectified Linear Unit) activation function and the Tanh (Hyperbolic tangent) activation function, and the discriminator uses the activation function of Leaky ReLU (Leaky Rectified Linear Unit). This approach has demonstrated excellent results, gradually making GAN networks controllable in the field of image generation (see Figure 1). Later, Isola et al. (2017) [6] delineated the possibility of GAN networks in the field of “style migration”. They first proposed the Pix2Pix network, which was not very mature initially. This network requires a certain similarity between the image in the source domain and the image in the target domain itself. Furthermore, the proposed CycleGAN (2017) [7] introduces the concept of an attention mechanism, which has become popular in the field of GAN networks.

Goodfellow et al. (2019) [8] proposed the concept of the adaptive GAN network model, a novel style transfer architecture that helps in learning normalized latent representation. Similarly, Brock et al. (2018) [9] proposed the BigGAN model, which improved the variety and stability of the images generated by the GAN model and reduced the model training time. Later, the multi-headed attention mechanism proposed by Daras et al. (2020) [10] solved the computational speed problem by sparsifying the attention feature map. In 2019, the ProGAN network model proposed by Gao et al. (2019) [11] made the fake image generation technology by the GAN networks more practical. They proposed a new training mechanism, starting from 4 × 4 images and gradually making the generated images more prominent, and proposed a smooth transition. This approach is more stable, and efficiently builds a GAN model that can generate high-quality large images. Recently, the StyleGAN (2019) [12] model proposed by NVIDIA has shown a mature face generation effect. This model inherits Gao’s idea and adds a style block module to control the image’s striking and obtained features. However, due to the “droplet artifact” problem in StyleGAN, Karras et al. (2020) [13] referred to the Multi-Scale Gradients-GAN (MSG-GAN) (2019) [14] to avoid the problem of over-representation of certain features during training by abandoning the fully connected layer and using a hopping approach. In this regard, the development of GAN networks in recent years has gradually gone from uncontrollable to controllable, from a single domain to cross-domain, and from small images to larger ones.

Due to the continuous development of GAN networks, generated virtual images have gradually become challenging to discern with human eyes alone, and can even have uses beyond humans. Recently, fabricated images of various avatars, celebrities, and even politicians have been released on many different media platforms, often while the audience is unaware of it [15], having a relatively negative influence on society. For example, the so-called “deepfake” is a technology that relies on the concept of deep learning technologies to create forged content. In 2018, comedian Jordan Peele made news when he posted a deepfake video of former U.S. President Obama insulting former U.S. President Trump, warning of the dangers of deepfake media [16]. Therefore, based on various contemporary technologies in the field of image recognition, researchers began to conduct considerable related research on this network, using features such as pixel points, frequency maps, co-occurrence matrices, manual feature extraction, and neural networks to construct detection tools leading to existing GAN models becoming more powerful. Although there are already many effective methods for attaining solutions, the problems of low accuracy and difficult generalization remain, leaving room for further improvement.

### 1.2. Problem Statement and Motivation

**(1) Vulnerability of existing models in realistic application scenarios**

The existing generative adversarial neural networks demonstrate excellent performance in detecting images generated by individual kinds of GAN networks; however, their major shortcoming is that the proposed models are generally very fragile. That is, if the method of generating fake faces (images) is replaced, then the detection performance of the whole model is drastically reduced and can even directly fail. This leads to the effectiveness of the detection model being drastically reduced in real-world settings.

**(2) Dataset limitations**

There are different ways to discuss such problems in general. For example, industrial-grade detection systems use millions of data items or even more as training sets to continuously improve the model, such as in target detection and image quality restoration. However, the resulting features of fake images generated by GAN networks may appear unrelated due to differences in the generation method used by the individual network. If the method uses large-scale datasets, obtaining results comparable to the general model is impossible. Therefore, the general nature of building a dataset of fake images is a challenging problem to overcome.

**(3) Difficulty in ensuring accuracy and generalization ability at the same time**

Marra et al. (2019) [17,18] considered the generalization problem by introducing the reinforcement learning method based on distillation learning. In this approach, after distillation of the neural network trained in one source domain, it is added to another target domain for re-training by retaining several key parameters that perform well in both domains. However, as the number of GANs is very large, the practical applications of this approach are limited. In other words, the difficulty in solving this problem is the existence of various GAN networks and complex feature extraction from fake images. Hence, it is not easy to maintain recognition accuracy while simultaneously maintaining generalization ability and robustness.

### 1.3. Contributions and Paper Outline

This research aims to develop a method under the umbrella of a one-class classification problem to successfully judge facial image authenticity. The contributions of this paper can be summarized as follows:

- We adopt Gaussian blur, Gaussian noise, and Homomorphic filter enhancement methods as basic pseudo-image generation methods for data enhancement.
- An improved Multi-Channel Convolutional Neural Network is used as the main network to accept multiple preprocessed data individually, obtain feature maps, and extract attention maps.
- Data are augmented using weakly supervised learning methods to add attention cropping and dropping to the data.
- The main network is trained in two steps, employing (a) a binary classification loss function to ensure that known fake facial features generated by known GAN networks are filtered out and (b) a one-class classification loss function to deal with different GAN networks or unknown fake face generation methods.
- A comparison of our proposed method against four recent methods in cross-domain and source-domain along with a numerical and graphical demonstration of the experimental results.

This paper consists of five parts. In Section 2, we introduce the technical background on which this paper is based, including the attention mechanism in WSL networks, the composition and structure of MCCNN, and the principle and application scenarios of the self-attention layer. Next, the overall proposed network architecture and the proposed improvements are disclosed. All the details are discussed in this section, including the training process and the data augmentation method of the Multi-Channel Convolutional Neural Network. A one-class classification model trained in two steps is presented here as well. In Section 3, the applied dataset used for training and testing is delineated, and the training environment of the device and the evaluation metrics are presented. Finally, Section 4 offers an overall summary of works related to this paper, introduces the future development and exploration of the topic and related research, and lists the main current problems in the field.

## 2. Proposed Methods

This section introduces the method proposed in this paper in detail. In Section 2.1, we discuss the background knowledge of the techniques proposed in this paper. First, the technical background of the effective data augmentation method is introduced. This can be used to address the shortcomings of the fake face dataset and avoid being limited to local features. Next, we describe the MCCNN that we use to increase the model’s robustness and discover the different features that may appear under different treatments. Section 2.2 introduces the process and structure of the proposed MCCNN-based discriminant model in this paper. Section 2.3 provides a detailed explanation of the data augmentation and application methods based on WSL. Section 2.4 discusses the selection of the proposed methods for improving the accuracy of the final model and assisting in feature extraction. Finally, Section 2.5 introduces the generation method of the entire model, which is a one-class model using two loss functions.

### 2.1. General Definition and Technical Background

#### 2.1.1. Weakly Supervised Learning

In order to prevent the occurrence of over-fitting and to further improve the overall performance of deep learning training models, data augmentation is generally used to increase the number of features as well as the variability of the dataset [19,20]. Furthermore, data augmentation methods are often applied in the computer vision domain, e.g., cropping, flipping, scaling, and Gaussian noise, respectively [21]. The principles of their respective roles are as follows.

**Cropping/random cropping**: one of the parts from the original data image is randomly cropped, and after the sample crop is then expanded to the size of the original data image for data input [22].

**Flipping**: rotates the image to a certain angle, with the center as the axis.

**Scaling**: similar to cropping in visual effect, the image is enlarged or reduced in proportion and then intercepted or enlarged in a certain area.

**Gaussian noise**: a noise class in which the probability density function follows a Gaussian distribution. Neural networks may train meaningless high-frequency features, leading to overfitting phenomena. The method of artificially adding noise is adopted to distort the image artificially. Gaussian noise is one of the more common methods of this type [23].

These conventional data augmentation methods often use a random approach to the data augmentation mechanism. The ”Weakly Supervised Data Augmentation Network (WS-DAN)” proposed by Hu et al. [24,25] introduces the idea that Weakly Supervised Learning (WSL) can be used to generate an “attention map” that indicates the salient features of the target to be detected/recognized during training. This generated “attention map” is later used in the subsequent training to perform targeting data augmentation.

#### 2.1.2. Bilinear Attention Pooling

The method proposed by WS-DAN for extracting attention maps is called Bilinear Attention Pooling (BAP). This method is based on the principle of bilinear pooling [25]. This is the most fundamental module among WS-DAN and the raw material used for subsequent operations. The key strategy of BAP is to obtain the feature map and the attention map through the network backbone. The attention map’s main part delineates the target’s specific part, which is the coordinates of the main feature. The corresponding distribution of feature maps can be obtained using the dot product of the elements of the attention map and the feature maps. The method is summarized in Equation (1):

(1) $$F_{fea_{n}}=F_{att_{n}}⊙F_{fea}\;\left(n=1,2,\ldots ,Q\right)$$

where $F_{fea}$ represents the feature map and $F_{att}$ represents the attention map. The ⊙ product delineates element-wise multiplication. Suppose there are a total of *P* feature maps and *Q* attention maps, resulting in the *n*th part feature map.

Afterward, an additional feature extraction function K(•) is required to extract the obtained local features, such as the global average pooling, global max pooling, or direct convolution. Thus, the attention feature corresponding MAP to the *n*th can be obtained, which is the so-called *Q-dimensional* vector. It is summarized in Equation (2):

(2) $$MAP_{n}=K\left(F_{fea_{n}}\right)$$

Finally, the feature maps of each division are combined to obtain the feature matrix of our target, which is calculated using Equation (3):

(3) $$T=\lambda \left(F_{att},F_{fea}\right)=\left(\begin{array}{c} K\left(f_{att_{1}}⊙F_{fea}\right)\\ K\left(f_{att_{2}}⊙F_{fea}\right)\\ \ldots \\ K\left(f_{att_{Q}}⊙F_{fea}\right) \end{array}\right)=\left(\begin{array}{c} PAF_{1}\\ PAF_{2}\\ \ldots \\ PAF_{Q} \end{array}\right)$$

PAF is the part of feature map $\lambda (F_{att},F_{fea})$, denoting the final generated bilinear attention pool, which is the discriminative feature *T* computed by the discriminative model for the object; *T* is an PQ-dimensional vector.

#### 2.1.3. Attention Regularization

Thanks to the mutual integration of multiple feature maps, this approach avoids the mutual influence caused by completely different features for the same object in the same situation. Thus, it is necessary to ensure that the features extracted in this case are the same. A loss function based on center loss [26] is used here, as illustrated in Equation (4); $P_{c}$ is the global feature center, and the loss function is denoted as $L_{att}$:

(4) $$L_{att}=\sum_{n=1}^{Q}{∥PAF_{n}-P_{c_{n}}∥}_{2}^{2}$$

Here, $P_{c_{n}}$ can be initialized to 0, and the value is updated using the Equation (5), while δ controls the update rate of $P_{c_{n}}$:

(5) $$P_{c_{n+1}}=P_{c_{n}}+\delta \left(PAF_{n}-P_{c_{n}}\right)$$

#### 2.1.4. Coordinate Location of Essential Features Based on Feature Attention Map

In image recognition, especially in deep learning, background noise other than the target to be recognized can significantly impact the final result. In addition, there is a need to avoid making the features one-sided due to an inevitable feature of a class of data being too prominent. The random attention maps generated by BAP can be defined as $F_{A}$ to guide the data augmentation. Later, this attention map is normalized. The normalization process summarized in Equation (6):

(6) $$F_{A_{n}}^{\#}=\frac{F_{A_{n}}-\min\left(F_{A_{n}}\right)}{\max\left(F_{A_{n}}\right)-\min\left(F_{A_{n}}\right)}$$

**Figure 2:**
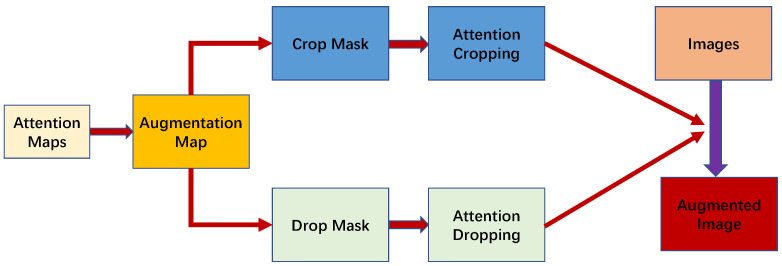
The architecture of Attention Guide [25].

Figure 2 illustrates the architecture of the Attention Guide (AG). For the attention feature maps generated by BAP, we performed two operations: Attention Cropping and Attention Drop.

We processed $F_{A_{n}}^{\#}$ in two ways. The first was used to obtain the crop mask directly from $F_{A_{n}}^{\#}$, which was calculated using Equation (7). Then, we used a minimum bounding box to cover all the ones in the $M_{CROP}$ and $M_{DROP}$ masks. Next, we went through the bounding box to crop the training data. After that, the training data were scaled to the original data size in order to clarify the more critical information. This process is known as attention cropping, where $\eta_{crop}$ is the set threshold:

(7) $$M_{CROP_{n}}\left(i,j\right)=\left\{\begin{array}{cc} 1, & F_{A_{n}}^{\#}\left(i,j\right)>\eta_{crop}\\ 0, & \;\mathrm{otherwise}.\; \end{array}\right.$$

We masked the most prominent feature coordinates in order to explore other features. The calculation method is summarized in Equation (8). This process is the so-called attention dropping:

(8) $$M_{DROP_{n}}\left(i,j\right)=\left\{\begin{array}{cc} 0, & F_{A_{n}}^{\#}\left(i,j\right)>\eta_{drop}\\ 1, & \;\mathrm{otherwise}.\; \end{array}\right.$$

The main scenario of attention dropping deals with the situation where one feature of a data is too prominent, and multiple attention maps may only pay attention to the same coordinate where the feature is located. Attention dropping can significantly alleviate this problem.

#### 2.1.5. Multi-Channel Convolutional Neural Network

**Figure 3:**
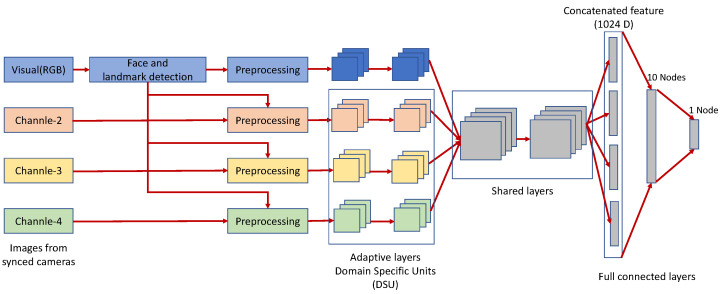
Architecture of Multi-Channel Convolutional Neural Network (MCNN) [27].

George et al. [27] proposed a novel Multi-Channel Convolutional Neural Network architecture based on LightCNN [28] for problems related to portrait detection. This network architecture addresses the inability to train a deep architecture from scratch when the dataset is limited. A proposed multi-channel neural network that utilizes a pre-trained face recognition model is thus constructed. The structure of the network is illustrated in Figure 3.

Unlike the common intention of uncovering high-level features for a particular task, Pereira et al. [28] proposed an approach where the shared high-level features are heterogeneous and retrained only for the lower layers. They delineate in their work that the parameters of the CNN can be segmented, with a part shared among different channels as higher-level features and only the lower-level features (Domain-Specific Elements) adjusted. Furthermore, George et al. [29] improved on this by choosing not to make the information representation of multiple channels the same, instead choosing to obtain complementary information jointly obtained by multiple channels. The features from different channels can then be linked; the decision boundaries are represented by backpropagation using a fully connected layer.

This MCCNN model is designed based on the LightCNN model. LightCNN [28] has the characteristics of using fewer parameters and pre-trained face features. LightCNN does not use the common ReLU activation function, instead employing the Max-Feature Map (MFM) [30] operation to suppress low-activation neurons in each layer. LightCNN uses a pre-trained model on face data expanded to four channels for data reception. After the features from multiple channels are convolved and mixed using Domain Specific Units (DSU), they are input to a fully connected layer using a sigmoid activation function for final feature extraction. The fully connected layer is adjusted for the problem of faces. In cases involving a large amount of data, the weights of the pre-trained face recognition network can be used. The goal is to deal with possible overfitting problems. The fully connected layer is then divided into two layers; the first layer has ten nodes, and the second layer has only one node.

### 2.2. Architecture of the Proposed Network

**Figure 4:**
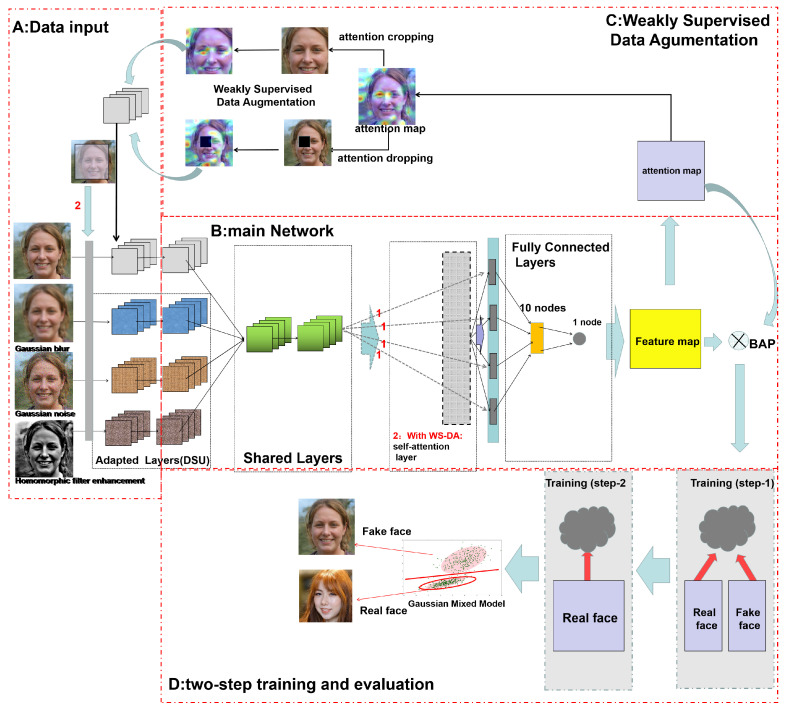
The architecture of the whole network. In this figure, the fake face is from the open-source StyleGAN dataset and the real face is from the open-source FFHQ dataset.

The overall architecture of the proposed network is shown in Figure 4. The basic structure of the network is inspired by Multi-Channel Convolutional Neural Network (MCCNN) [27]. It includes a data augmentation module based on WSL and a one-class classification module taking into account two training sessions: (a) binary classification and (b) one-class classification, as well as the basic training process of the original network.

It is divided into four blocks.

**Block A**—data input represents the input of the entire network.

**Block B**—the main network, which is the core structure of the network, including the convolution pooling layer and the fully connected layer.

**Block C**—weakly supervised data augmentation, which shows the flow of the data augmentation method utilizing attention mapping.

**Block D**—a two-step training and evaluation process; the single-class discrimination model is generated using the two-class loss function and the single-class loss function together to fix the features.

In this paper, we introduce a weakly supervised learning mechanism for fine-grained classification to discover the apparent differences between real and fake faces, considering the difficulty of generalization and building a complete dataset for the problem of fake faces generated by GANs. Furthermore, we connect the binary classification problem of recognizing real and fake faces with the traditional portrait anomaly detection problem [29]. Known fake face types and unknown fake face types are sliced using the training of the two-category classifier and the one-class classifier. Here, the two categories are True and NotTrue; the NotTrue category includes the fake face category and the unknown category. Finally, a feature model of the real faces generated by the GAN is finally obtained and a traditional judgment based on probability is applied; if the probability is greater than 50%, a face can be judged as True.

As shown in BlockB in Figure 4, the proposed MCCNN is a multi-channel convolutional neural network that can accept multiple pre-processed data separately for feature recognition. This network convolves the features of each channel separately using Domain-Specific Elements (DSEs), then sums the features with complementary properties. At first, the features are directly fed to the fully connected layer for feature fixation and the feature map is obtained. Later, the attention map is extracted and the data are augmented using WSL methods to add cropping and dropping attention maps into the initial training data.

Moreover, in BlockC, the data are augmented using WSL, and the cropping attention map and dropping attention map are added to the initial training data. Finally, in BlockD, we employ the self-attention layer to construct pixel–dot connections and then add the features to the fully connected layer after obtaining the complementary features in this training. After obtaining the final features, we use the loss function of binary classification and the loss function of one-class classification for training. First, the known fake face features generated by known GANs are excluded, then the real face features are aggregated to deal with other types of GANs or unknown false face generation methods.

This approach generates a discriminative model that allows the detection of false faces generated by GANs without limiting the features of a particular GAN and can be separated from the false face dataset by discovering the “differential features” of real faces to continue to determine the results. Therefore, it is possible to improve the generalization ability and reduce the vulnerability of the overall model while maintaining the accuracy of recognition of the source domain. Figure 4 explains the overall training process, data augmentation, and discriminative model generation. After these processes, a one-class classification model that improves generalization performance while maintaining accuracy is produced.

### 2.3. Data Augmentation and Preprocessing

Following BlockA in Figure 4, there are two main data augmentation and preprocessing modules, one of which is the primary data augmentation module. The key role of this module is to preprocess the original data image, increase the robustness of fake face recognition by manually adding noise to the original image, and try to avoid detecting only a single feature. We screened out the following five preprocessing methods based on previous research and experiments.

**Gaussian blur**: known as Gaussian smoothing, its primary generation method can be summarized as convolution with a normal distribution, a so-called low-pass filter for the image.

**Gaussian noise**: Gaussian noise is a kind of hand-added noise, which includes undulating noise, cosmic noise, thermal noise, and scattered grain noise. The probability distribution function of the noise follows a normal distribution.

**Motion blur**: known as motion blur, it stimulates the apparent traces of blurring and dragging when the image shows motion effects.

**Homomorphic filter enhancement**: homomorphic filtering acts in the frequency domain. Its role is to adjust the image’s grayscale range to enhance image detail by eliminating uneven illumination without losing image detail in bright areas. It can compress the image brightness range and enhance image contrast, and attempts to suppress the low-frequency energy, which reduces the dynamic range and increases the higher frequencies, enhancing the image’s contrast.

**Fourier transform (magnitude spectrum)**: Fourier transform mainly turns the signal in the time domain into the signal in the frequency domain, which is used for image noise reduction, enhancement, and other processing.

As summarized in Table 1, we used our models to screen each of these five preprocessing methods using ProGAN as the source domain and cross-domain StyGAN2 as the detection generalization performance. The five preprocessing methods are:

1. Gaussian blur
2. Gaussian noise
3. Motion blur
4. Homomorphic filter enhancement
5. Fourier transform (magnitude spectrum)

**Figure 5:**
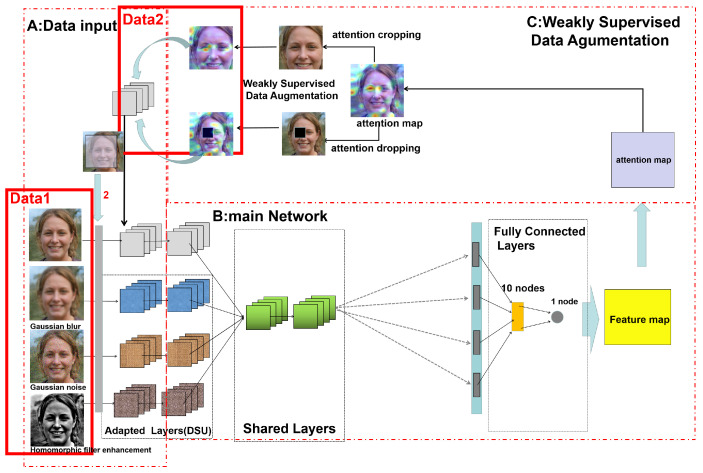
The process of data augmentation and preprocessing. In this figure, the fake face is from the open source StyleGAN dataset and the real face is from the open source FFHQ dataset.

The results disclosed in Table 1 demonstrate the accuracy rates (in percentages) of different combinations of preprocessing methods. Here, the combination of Gaussian blur, motion blur, and homomorphic filter enhancement has the highest accuracy rate, followed by the combination of Gaussian blur, Gaussian noise, and homomorphic filter enhancement and the combination of Gaussian noise, motion blur, and homomorphic filter enhancement, both for source-domain and cross-domain performance. Thus, we chose these three methods as our primary data augmentation method. The preprocessed data are labeled as DATA1 in Figure 5. After preparing DATA1 (which includes both preprocessed and original data), they were imported into the MCCNN model. Later, we obtained the attention map through basic training, which is shown in Figure 5. This part uses a basic MCCNN architecture; after the second fully-connected layer assigns the data weights, we get the feature map, which is the basis for the next step of data augmentation.

#### 2.3.1. Data Augmentation Based on Weakly Supervised Learning

As illustrated in Figure 5, for BlockC, the feature map is obtained from the fully connected layer. We define the obtained feature map as $F_{fea}$.

Following the idea proposed by CNN visualization [31], we obtained the coordinates for feature extraction. We take the forward calculation method and perform a 1×1 convolution operation for $F_{fea}$. The resulting process is illustrated using Equation (9):

(9) $$F_{att}=F\left(F_{fea}\right)=\overset{TOL}{\underset{Q=1}{⋃}}F_{att_{Q}}$$

where TOL represents the total number of generated attention maps, as the attention model is composed of multiple overlapping combinations of attention maps. This is proportional to the total number of attention maps for the accuracy of the whole model. According to Hu et al. [27], the accuracy of the whole model stabilizes when the number reaches 32; thus, we set TOL to 32. Then, we used the attention map to obtain the DATA2, as shown in Figure 5. Later, we performed attention cropping (sometimes known as interest domain selection) and attention dropping on the obtained attention map by setting a hyperparameter. If the value of a pixel is greater than this hyperparameter, it is changed to 1, and if it is less than this, it is changed to 0. The part that becomes 1 is the area to focus on. After obtaining this region, it is resampled to the original image size and used as an enhanced dataset for training. Attention dropping is used to erase this part from the image and use the rest as the training set to participate in the training of the model [25].

#### 2.3.2. Removing Low-Impact Influence Part

**Figure 6:**
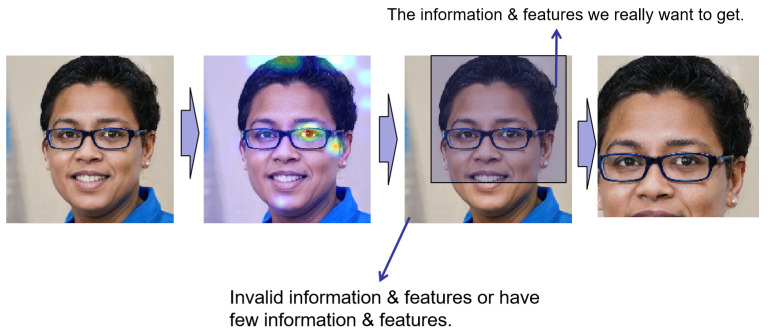
GAN network low-resolution to high-resolution feature mapping. In this figure, the fake face is from the open source StyleGAN2 dataset.

Hu et al. [27] proposed using the attention map to eliminate the influence of the low-impact part when using the attention map for fine-grained classification in object detection. As shown in Figure 6, with the information obtained by the attention map, we can obtain the concentrated area of the face to better discriminate between true and false information. We then can focus on this part of the area for training. As we have located the face and eliminated almost all the background information, we only select 80% of the parts for interception. After processing, most low-impact information on the face is eliminated. Then, the corresponding features are processed in the next step.

### 2.4. The Process of Training

**Figure 7:**
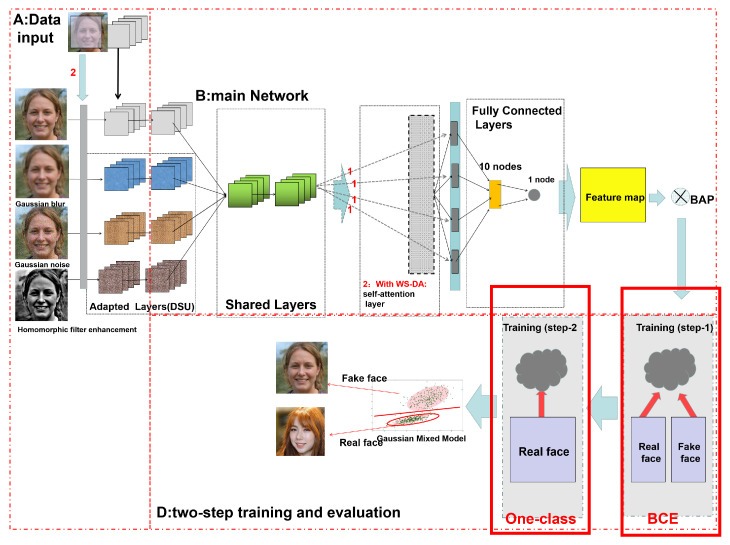
The process of training. In this figure, the fake face is from the open source StyleGAN dataset and the real face is from the open source FFHQ dataset.

We obtained the desired data through the basic training process of the MCCNN and data augmentation. After acquiring these data, we began the formal model training, with the underlying network architecture the same as that used for data augmentation based on the Weakly Supervised Attention (WSA) mechanism. The formal training process is illustrated in Figure 7. The two main points worth mentioning are (a) the inclusion of a self-attention layer to account for global features and (b) the training of a particular one-class classification model that borrows two loss functions together. These two points are introduced in detail in the following sections.

#### 2.4.1. Self-Attention Layer

As shown BlockB in Figure 7, after obtaining enough data and locating the feature coordinates of the image, we need to think about how to maximize the use of these data. There is another essential difference between the fake image generated by the GAN network and the real face image, namely, the difference in the overall texture features of the image.

**Figure 8:**
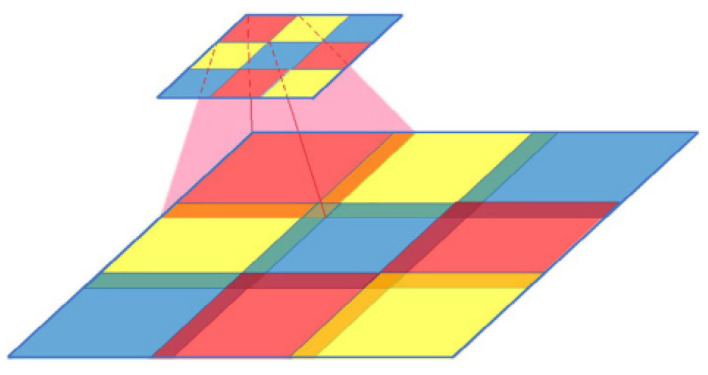
GAN network building images [29].

**Reason for using self-attention layer:**Figure 8 shows one of the weaknesses of contemporary GAN network-generated images, as summarized by Mi et al. [32]. They point out that during the development of GAN networks, the developers chose transposed convolution to provide the most suitable upsampling method for GAN networks [33]. However, such a sampling method has a severe problem because of a basic general generation process in the existing best GAN generation networks which requires re-upgrading a low-resolution image layer by layer and then expanding it into a high-resolution image. Figure 8 shows the relationship between small and large feature maps. We can find that the overlapping part is minimal and the non-overlapping part comes from learning separate local information, which has independent randomness. Therefore, the conclusion is that the images generated by GAN networks lack the global features of real images. In simple terms, it is not easy to distinguish an image generated by the GAN network from a real face image when viewed by human eyes, as it consists of many features from the real image in the local area. These local features are combined to deceive the human eye. Although Odena et al. [34] have proposed a number of methods to compensate for the lack of global features, it does not solve the fundamental problem. The global features are a weakness of GAN network generation that can be an essential reference for detection.

**Figure 9:**
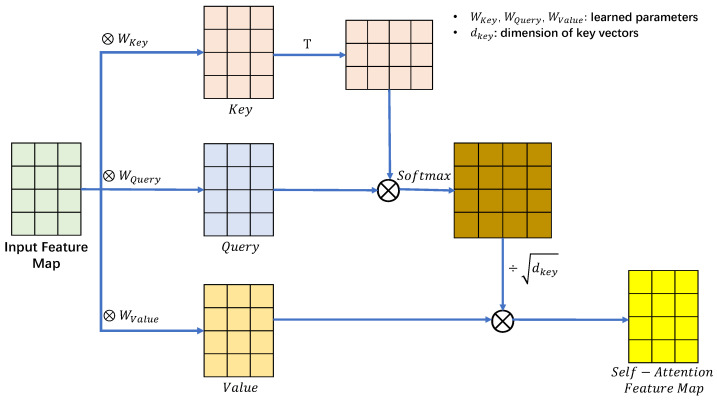
The operating principle of self-attention [29].

**Principle of self-attention layer:**Figure 9 shows the basic structure of the self-attention layer. The essence of the attention mechanism is to provide a focus similar to that of humans in machine learning rather than using a single machine cycle in the training process. Vaswani et al. [35] originally proposed the application of a self-attention layer in the field of natural language to allow the model to take into account the overall meaning of a sentence rather than being limited to only the relationship between adjacent words. The convolutional neural network’s convolutional kernel covers a minimal area, and it is only by stacking multiple convolutional layers that the perceptual field of the network can be expanded. This has the same limitations as natural language processing, where only the relationships between adjacent words can be noticed at the beginning. The purpose of using the self-attention layer is to consider the relevance of any two pixels in the image, allowing the global features of the whole image to be obtained.

In Equation (10), *n* is the coordinate of the calculated pixel point. The operation matrix is the same Query, Key, and Value as used for attention [35]. The uniqueness of the self-attention layer is that Query and Key are extracted from a sample pool. By placing the Query into the original sample, comparing with all the keys, and then comparing the similarity, the ultimate goal is to obtain the correlation value of all the pixels to the selected pixels in the feature map. Here, Wei is the weight matrix and $F_{fea_{n}}$ is the features of pixel *n*.

(10) $$\begin{array}{cc} Que_{n} & =F_{fea_{n}}\cdot Wei_{que}\\ Key_{n} & =F_{fea_{n}}\cdot Wei_{key}\\ Val_{n} & =F_{fea_{n}}\cdot Wei_{val} \end{array}$$

The attention $F_{att}$ can be obtained by normalizing and weighting the sum of the correlation values among all combinations using Equation (11):

(11) $${F_{att}}_{n}^{m}=\mathrm{softmax}\left(E\right)=\frac{Standardize\left(Que_{n}\cdot sk_{t}^{T}\right)}{\sum_{t}Standardize\left(Que_{n}\cdot sk_{m}^{T}\right)}$$

Then, we can obtain the global features $F_{OAfea}$ using Equation (12):

(12) $$F_{OAfea}=\sum_{t}{F_{att}}_{n}^{t}\cdot Val_{t}$$

This approach treats all the effective pixels as neighboring pixels after summarizing them, in other words, after reducing most non-active pixel points. However, the problem is broken by using global features that are difficult to focus on in GAN networks.

#### 2.4.2. Two-Step Training of One-Class Classification

Due to the variety of GANs and the speed of update iteration (see BlockD), it is difficult to improve the robustness of the whole model even if it has good performance for the source domain dataset, which is insufficient to generate images in practical applications with a large number of rapidly updated GANs. Therefore, we consider the one-class classification method here.

**Binary Classification Loss–Binary Cross-Entropy:** In this step, we use the BAP to combine the feature map and the attention map to generate the feature matrix, then use the loss function to fix the weights for training. The first is the most traditional loss function for binary classification, namely, binary cross-entropy, which is calculated by Equation (13), with positive samples labeled as 1 and negative samples labeled as 0:

(13) $$Loss_{BCE}=-\left(y\log\left(F_{pos}\right)\right)+\left(1-y\right)\log\left(1-F_{pos}\right)$$

where *y* is the dataset marker (marked as 1 when it is an accurate picture and 0 when it is a false picture), $F_{pos}$ is the probability of both occurring, and the sum of positive and negative samples is 1.

**One-Class Loss Function:** In this step, following the ideas of Wen et al. [26] and Hadsell et al. [36], a loss function is summarized that considers both the distance within a class and the distance between classes (called the OCCL) [29]. The main principle is to concentrate on a certain class by increasing the distance between classes in the loss function.

The formula for the loss function can be expressed by Equation (14), where $F_{wei}$ denotes the weight, $F_{lab}$ is the label of the selected class target, *P* is the selected operation’s target, $F_{disPC}$ is the distance from the target to the center, $Len_{line}$ is the margin, $P_{C}$ is the coordinates of the center, λ is a scalar that prevents changes in the center of the class on a small scale, and *n* is the displacement from the last *batchPc* that occurred during the training process:

(14) $$\begin{array}{cc} Loss_{OCCL}\left(F_{wei},F_{lab},P\right)= & F_{lab}\frac{1}{2}F_{disPC}^{2}+\left(1-F_{lab}\right)\frac{1}{2}\max{\left(0,Len_{line}-F_{lab}\right)}^{2} \end{array}$$

(15) $$\begin{array}{cc} F_{disPC}= & \sqrt{{∥P^{i}-P_{C}∥}_{2}^{2}} \end{array}$$

(16) $$P_{C}={\hat{P}}_{C}\left(1-\lambda \right)+\lambda \frac{1}{N}\sum_{i=1}^{N}n_{i}$$

### 2.5. Final Model–Gaussian Mixture Model

The final loss function used to fix the training weights is illustrated by Equation (17); here, θ is set to 0.5:

(17) $$Loss=\left(1-\theta \right)Loss_{BCE}+\theta Loss_{OCCL}$$

After this, we build the final classification model using the Gaussian mixture model. The Gaussian mixture model combines *M* multivariate single Gaussian models. This mixture model has good mathematical properties and excellent computational performance. Following [37], it can be expressed using Equation (18).

(18) $$F_{psum}\left(P|\Theta \right)=\sum_{I=1}^{I}F_{pI}$$

Here, *P* is the selected target, $F_{psum}$ is the probability of the overall model, $F_{p}$ is the probability of the individual Gaussian model, and Θ is the expectation, variance/covariance, and probability of occurrence in the mixture model for each sub-model. For the Gaussian mixed discriminant model, the parameters were chosen to maximize the expectation and five objectives were chosen, that is, *I* = 5 for each objective for the covariance matrix. The calculation of scores selects the log-likelihood score using Equation (19), as summarized below:

(19) $$F_{sco}=\log\left(F_{psum}\left(P|\Theta \right)\right)$$

## 3. Datasets, Experiments, and Results

This section mainly discusses the proposed model’s performance using two sets of experiments. *Experiment 1* aims at the essential discrimination accuracy and evaluates the model’s performance for the source domain (the same type of data as the training data set). *Experiment 2* tests the model’s generalization ability and then measures and compares its detection ability for cross-domain data.

Section 3.1 illustrates the types and characteristics of the datasets used. In Section 3.2, the experimental setting and parameters are discussed. Section 3.3 delineates a rough introduction of the recent methods for making such comparisons and presents the experimental results, followed by a summary. Finally, Section 3.4 summarizes the findings and conclusions of the whole experiment.

### 3.1. Dataset

The datasets used in the experiment are public datasets and data generated by GANs, as described in Section 1.1.

**Figure 10:**
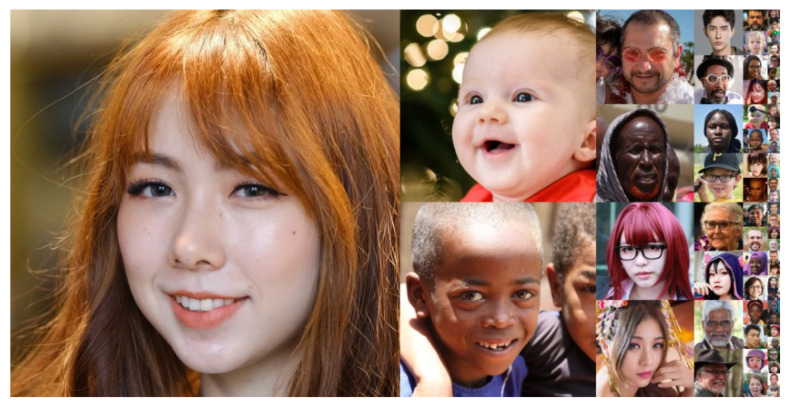
FFHQ Dataset [12].

**Flickr-Faces-High-Quality (Flickr-Faces-HQ)**: This dataset [12] itself was created as a benchmark for coping with GANs. NVIDIA has made it open source for the community since 2019. It incorporates 70,000 high-definition face images in PNG format with a resolution of 1024 × 1024. Among the differences are age, race, and image background. As shown in Figure 10, the attributes of faces include age, gender, race, skin color, expression, face shape, hairstyle, face pose, with or without eyes (i.e., sunglasses), hats, hair accessories, scarves, etc., for a full range of variations. This database is widely used for classifying face attributes or semantic segmentation models of faces. It is sourced from Flickr by crawling, uses dlib for face alignment and cropping, and removes non-real face images such as statues.

**ProGAN**: A model proposed by Gao et al. (2019) [11]. NVIDIA uses ProGAN to generate images with 1024 × 1024 resolution. The GAN model generates efficient and stable images gradually growing larger in size.

**StyleGAN**: This GAN model proposed by NVIDIA in 2019 is based on ProGAN [12], which removes the input layer, takes the model of a nonlinear mapping network as an input, and enhances it by first generating eye-catching features and then generating detailed features. The resolution is 1024 × 1024 pixels.

**StyleGAN2**: NVIDIA’s latest proposal to address the shortcomings of StyleGAN. In StyleGAN2 [14], they modify the generator architecture and the sample normalization, thus removing the artificial traces and making the generation results more controllable. The resolution is 1024 × 1024 pixels.

**BigGAN**: DeepMind proposed BigGAN [9] in 2018, with the primary goal of closing the gap in fidelity and diversity between images generated by GANs and real-world images from the ImageNet dataset. BigGAN trains generative adversarial networks at the most significant scale and investigates the instabilities specific to this scale. The resolution is 1024 × 1024 pixels.

**DeepFake**: In late 2017, a Reddit user named “deepfakes” [38] used celebrity faces to generate pornographic video conversions and posted them on the web. This quickly spread across media and networks, and many new deepfake videos have begun appearing. The resolution is 1024 × 1024 pixels.

**DCGAN**: DCGAN is a combination of a deep convolutional network and GAN proposed by Radford et al. (2015) [5]. It mainly replaces the generative and discriminative networks with two convolutional networks (CNN). The purpose is to improve the quality of the generated samples and the convergence speed of the network. The resolution is 1024 × 1024 pixels.

**VQ-VAE 2.0**: In response to the possible problems of mode collapse and insufficient diversity in BigGAN, DeepMind proposed the VQ-VAE-2 generative model (2019) [39]. The variational autoencoder “VAE” is an unsupervised learning method that belongs to a robust variant of AutoEncoder.

### 3.2. Environment Settings

The operating system of our experiments was Ubuntu 18.06, and the device’s CPU was an Intel(R) Core(TM) i9-10940X CPU@3090GHZ. The GPU used in this work was an NVIDIA Corporation GA102 (GeForce RTX 3090). The variable values of the CNN network were referred to as LightCNN [28]. The size of all images was set to 256 × 255. The nodes of the fully connected layer were set to 10 and 1, and the variable θ that assigns weights to the two loss functions was set to 0.5. Furthermore, the training data were randomly selected from the original dataset and the test data were randomly selected from the remaining data. After several trials and errors, 50, 100, 150, 200, 250, 350, 500, and 250 were selected as epochs. Each set of experimental data was tested seven times, then the average of the middle five times was obtained after removing the best and worst performances.

### 3.3. Experiments and Results

#### 3.3.1. Experiment 1: Scrutinizing Performance of the Source Domain

**Experimental parameters:** As summarized in Table 2, we examined the performance of the recent methods in the source domain (FFHQ, ProGAN). Referring to [40], a standard selection of measures was used, namely, (a) accuracy: the percentage of data that correctly classify fake and real faces in the entire test dataset; (b) precision: the proportion of ground truth real faces among all predicted real faces; (c) recall: the proportion of data successfully predicted as a real face in all ground truth real face data; and (d) F1-Score: the harmonic mean of precision and recall for each method for evaluation. For this experiment, we trained the model with 12,000 real faces from FFHQ and 12,000 fake faces from ProGAN with resolution 1024×1024, with the images bilinearly interpolated and transformed to 256×256 size. The test was conducted with 2000 real faces from FFHQ and 2000 fake faces from ProGAN with the resolution of 1024×1024, and the images were bilinearly interpolated and converted to 256×256 size.

**Experimental results:** The obtained results show that our detection accuracy for real face images (FFHQ) ranks second overall with 0.994, slightly lower than the Pupil regular recognition+Boundary IoU score (2022). Furthermore, for fake face detection in the source domain, our method ranks at the top with 0.989, and the F1-Score of our method ties for first with the Pupil regular recognition+Boundary IoU score (2022) method with 0.992.

**Summary of Experiment 1:** Although our method does not have excessive performance differences in the source domain compared to recent methods, the detection accuracy of fake face images is slightly better than the other methods. Among them, the results of Pupil regular recognition+Boundary IoU score (2022) are better than ours, though the gap is minimal. Accuracy is a separate test on this dataset, and other statistical measures are the results obtained by randomly mixing and identifying the data of real and fake faces.

#### 3.3.2. Experiment 2: Scrutinizing Cross-Domain Performance

**Experimental parameters:** We tested the performance of our method in cross-domain detection against recent methods (e.g., StyleGAN, StyleGAN2, BigGAN, DCGAN, DeepFake, VQ-VAE2.0). For each method, we measured the accuracy, precision, recall, and F1-score for evaluation. In this experiment, we trained the model with 12,000 real faces from FFHQ and 12,000 fake faces from ProGAN with resolution 1024×1024, with the images bilinearly interpolated to 256×256 size. The test uses a fake face dataset of 2000 images, each generated by the StyleGAN, StyleGAN2, BigGAN, DCGAN, DeepFake, and VQ-VAE2.0 models; the resolution of images are 1024×1024, and the images are bilinearly interpolated and transformed to 256×256 size.

**Experimental results:** The performance results on cross-domain datasets are demonstrated in Table 3 and Table 4. Among the methods similar to the ProGAN principle, the accuracy of StyleGAN reached 0.872, and the corresponding F1-Score reached 0.937. The accuracy of StyleGAN2 reached 0.851, and the F1-Score achieved 0.928. Among the other four kinds of GAN networks, the worst performance is demostrated by VQ-VAE2.0, with an accuracy of 0.809 and an F1-Score of 0.910. The best performance is demonstrated by DCGAN, with an accuracy of 0.931 and an F1-Score of 0.964.

For data comparison results, the accuracy of our proposed method in cross-domain detection is improved by more than 30% against the CNN+Self-attention (2020) method. Although the Pupil regular recognition+Boundary IoU score (2022) method performs equally well, it has similar results to ours. The worst performance is demonstrated by VQ-VAE2.0, with an accuracy of 0.722, which shows that its performance does not remain stable when faces are generated using various GANs with different principles. This is because it relies on the instability of GANs for the generation of human eyes, and this instability has different effects on the fake faces generated by different types of GANs.

**Summary of Experiment 2:** Following the experiments, we can conclude that our method has relatively good performance and stability compared to recent fake face detection methods, even considering cross-domain problems. Similar to Experiment 1, accuracy is a separate test on this dataset, and other statistical measures are the results obtained by randomly mixing and identifying the data of real and fake faces.

**Figure 11:**
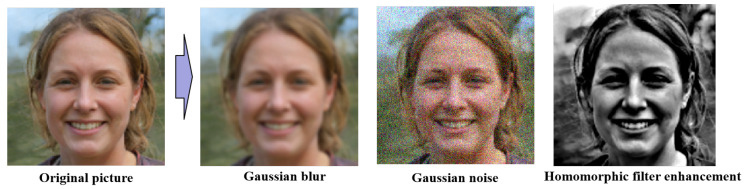
Face data preprocessing. In this figure, the fake face is from the open-source StyleGAN dataset.

In separate but related work, we performed three types of preprocessing on the images in the initial data preprocessing stage, corresponding to the four input pipelines of MCCNN, as shown in Figure 11.

**Figure 12:**
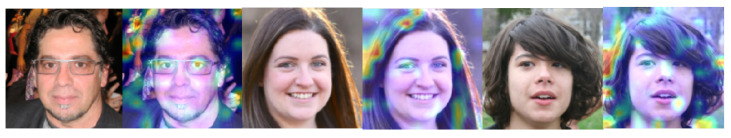
Face images detected as real. In this figure, the real face is from the open-source FFHQ dataset.

**Figure 13:**
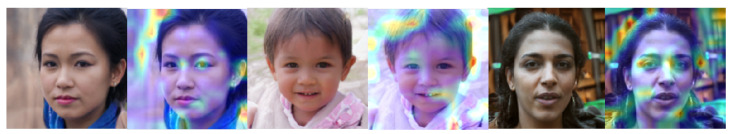
Face images detected as fake. In this figure, the fake face is from the open-source StyleGAN dataset.

Figure 12 and Figure 13 demonstrate detection cases. The feature distinction between real and fake images is concentrated on the hair, eyes, and bridge of the nose and mouth.

**Figure 14:**
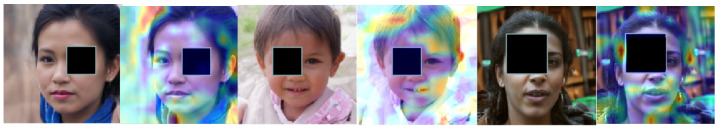
Face images detected as fake (attention change). In this figure, the fake face is from the open-source StyleGAN dataset.

Figure 14 shows the effectiveness of tuning attention for other feature discoveries. More areas of the face, forehead, etc., are considered.

### 3.4. Findings

**Source domain performance:** Our proposed method maintains acceptable accuracy close to other recent methods on the source domain, and performs better on fake images. When the test data use the same data as the training data, the identification of real images in FFHQ with the detection result reaches 99.4%, slightly lower than the Pupil regular recognition+Boundary IoU score (2022) method. Nonetheless, it maintains an excellent level among the detection methods. Next, for detecting the fake face images in the source domain, which are the data from the ProGAN dataset, our accuracy rate reaches 98.9% compared to Pupil regular recognition+Boundary IoU score (2022), which demonstrates 98.8%. The F1-Score of our method, at 0.992, outperforms the selected recent detection methods.

**Cross-domain performance:** The cross-domain performance of the method proposed in this paper is dramatically improved compared to the original detection method, though there is room for improvement in practical application. We employed StyleGAN, StyleGAN2, BigGAN, DCGAN, DeepFake, and VQ-VAE2.0 as our unknown fake face generation methods, and used FFHQ as the real default face. For cross-domain detection performance, the accuracy of our proposed method exhibits 87.2% on StyleGAN, 85.1% on StyleGAN2, 82.7% on BigGAN, 93.1% on DCGAN, 89.7% on DeepFake, and 80.9% on VQ-VAE2.0, respectively. Compared to other recent detection methods, especially methods that do not consider generalization ability, our accuracy improves on them by 5% to 30% and in F1-Score by 105% to 205%.

## 4. Conclusions and Future Work

### 4.1. Conclusions

This paper proposes a one-class classification model for detecting fake faces generated by various GAN networks. It aims at the problems of high vulnerability in fake face detection and difficult cross-domain detection. The model mainly focuses on exploiting the avoidance of extracting only local data features and fine-grained extraction of existing features. In addition, this research deals with images of human faces, and explores a method to distinguish whether the image is a real face or a fake artificial image. All facial image data used here were collected from public datasets. We do not authenticate that an image is a specific individual from the face image, nor do we identify individuals from the face images. Therefore, there are no ethical issues in this study.

**(1)**  **Method and main process of the model**

The proposed approach first uses the basic Gaussian blur, Gaussian noise, and homomorphic filter enhancement methods for data augmentation to increase the model’s robustness. In the first round of feature extraction, a WSA mechanism was used to enhance the data by modifying the key regions of the model discriminator. Then, we removed the low-impact information with the help of the obtained attention map. After all the data were obtained, they were used as the input of multiple channels of an MCCNN. A self-attention layer was employed to build the connection between pixels, improving the recognition accuracy. The final discriminative model was generated by employing (a) a Binary Cross-Entropy loss function, (b) one-class classification loss among the fixed features, (c) two loss functions, and (d) the Gaussian mixture model. Then, the final features of the real face was obtained.

**(2)**  **Experiments and evaluation**

To test cross-domain and source domain performance separately, in the experiments we compared our proposed method to four recently published methods. We used real face images from FFHQ and fake face images from ProGAN as source data for system training and used ProGAN as a known fake face generation method against unknown fake face generation methods such as StyleGAN, StyleGAN2, BigGAN, DCGAN, DeepFake, and VQ-VAE2.0. The model’s performance was judged following standard statistical measures, e.g., accuracy, overall accuracy, precision, recall, and F1-score. Through our experiments, it is confirmed that the proposed method performs well in both the source domain and cross-domain.

### 4.2. Future Work

There are two key points for further consideration in future works.

**(1)**  **Considerations focusing on one certain facial feature**

Although we have tried very hard to consider the influence of removing the background and other irrelevant factors on the results, nonetheless, the different amounts of information contained in different parts of the face are a significant problem in the field of fake face detection.

**(2)**  **GANs considering different principles**

Our experiments show that ProGAN, StyleGAN, and StyleGAN2 are based on the same principle. Although the model has been upgraded, our detection accuracy remains stable. However, for other GAN networks with completely different principles, the accuracy rate fluctuates to a certain extent. The solution to this problem is a topic for future work that needs to be considered as a next step.

## Figures and Tables

**Table 1:** Results of different combinations.

Combination	ProGAN	StyleGAN2
1 + 2 + 3	98.3%	82.4%
1 + 2 + 4	98.5%	83.7%
1 + 2 + 5	98.3%	82.1%
1 + 3 + 4	98.9%	85.1%
1 + 3 + 5	98.3%	80.9%
1 + 4 + 5	98.2%	83.4%
2 + 3 + 4	98.4%	84.1%
2 + 3 + 5	95.1%	81.9%
2 + 4 + 5	98.3%	82.4%
3 + 4 + 5	98.5%	82.1%

**Table 2:** Result 1: performance of the source domain.

Method		FFHQ	ProGAN
Ours	Accuracy	0.994	0.989
	Precision	0.990	
	Recall	0.994	
	F1-Score	0.992	
CNN+Self-attention (2020)	Accuracy	0.991	0.974
	Precision	0.984	
	Recall	0.991	
	F1-Score	0.987	
Pupil regular recognition+Boundary IoU score (2022)	Accuracy	0.995	0.988
	Precision	0.988	
	Recall	0.995	
	F1-Score	0.992	
Dual-color spaces+improved Xception model (2021)	Accuracy	0.994	0.981
	Precision	0.981	
	Recall	0.994	
	F1-Score	0.988	
MaskCNN+RAN (2022)	Accuracy	0.989	0.975
	Precision	0.975	
	Recall	0.989	
	F1-Score	0.982	

**Table 3:** Results 2.1: cross domain performance.

Method		StyleGAN	StyleGAN2	BigGAN
Ours	Accuracy	0.872	0.851	0.827
	Precision	0.886	0.870	0.852
	Recall	0.994	0.994	0.994
	F1-Score	0.937	0.928	0.917
CNN+Self-attention (2020)	Accuracy	0.514	0.497	0.551
	Precision	0.671	0.663	0.688
	Recall	0.991	0.991	0.991
	F1-Score	0.800	0.795	0.812
Pupil regular recognition+ Boundary IoU score (2022)	Accuracy	0.861	0.754	0.817
	Precision	0.878	0.802	0.845
	Recall	0.995	0.995	0.995
	F1-Score	0.933	0.888	0.914
Dual-color spaces+improved Xception model (2021)	Accuracy	0.769	0.728	0.657
	Precision	0.811	0.785	0.743
	Recall	0.994	0.994	0.994
	F1-Score	0.893	0.877	0.851
MaskCNN+RAN (2022)	Accuracy	0.507	0.501	0.751
	Precision	0.667	0.665	0.799
	Recall	0.989	0.989	0.989
	F1-Score	0.797	0.795	0.884

**Table 4:** Result 2.2: cross domain performance.

Method		DCGAN	DeepFake	VQ-VAE2.0
Ours	Accuracy	0.931	0.897	0.809
	Precision	0.935	0.906	0.839
	Recall	0.994	0.994	0.994
	F1-Score	0.964	0.948	0.910
CNN+Self-attention (2020)	Accuracy	0.412	0.532	0.461
	Precision	0.628	0.679	0.648
	Recall	0.991	0.991	0.991
	F1-Score	0.769	0.806	0.783
Pupil regular recognition+ Boundary IoU score (2022)	Accuracy	0.921	0.874	0.722
	Precision	0.926	0.888	0.782
	Recall	0.995	0.995	0.995
	F1-Score	0.959	0.938	0.875
Dual-color spaces+improved Xception model (2021)	Accuracy	0.834	0.787	0.614
	Precision	0.859	0.824	0.720
	Recall	0.994	0.994	0.994
	F1-Score	0.920	0.901	0.835
MaskCNN+RAN (2022)	Accuracy	0.745	0.671	0.719
	Precision	0.795	0.750	0.779
	Recall	0.989	0.989	0.989
	F1-Score	0.881	0.853	0.871

## Data Availability

Not applicable.
